# Stature estimation from footprint measurements in Bangladeshi adults

**DOI:** 10.1080/20961790.2020.1776469

**Published:** 2020-07-02

**Authors:** Md Asadujjaman, Md Harun Or Rashid, Md Sohel Rana, Md Mosharraf Hossain

**Affiliations:** Department of Industrial & Production Engineering, Rajshahi University of Engineering & Technology, Rajshahi, Bangladesh

**Keywords:** Forensic sciences, forensic anthropology, anthropometry, footprint, stature estimation, forensic identification

## Abstract

The estimation of stature is very important in forensic investigation, as it provides useful data that can narrow the pool of potentially matching identities. The purpose of this study was to develop formulae for the estimation of stature from footprint measurements in Bangladeshi adults. This study included 118 randomly selected men and 130 randomly selected women, all aged 18–50 years. From each participant, stature and six footprint measurements were taken by means of standard measurement techniques. Footprint measurements were found to be positively correlated with stature. Stature was estimated by using linear regression equations. The right T1 length in men (*R:* +0.587, *R^2^*: 0.345) and the right T2 length in women (*R*: +0.506, *R^2^*: 0.256) were the most reliable individual estimators of stature. However, when data were combined for both sexes, the right T2 length was identified as the most reliable estimator of stature, with higher values of *R* (+0.792) and *R^2^* (0.627). In conclusion, human stature can be successfully estimated by using footprint measurements; this finding can be applied in forensic research and investigation.

## Introduction

The estimation of stature based on various human body parts is a common focus in forensic science and medicine, as well as in ergonomics and human factors engineering. Human body measurements have been used to identify criminals and victims in accidents, natural disasters, and terrorist attacks or as war casualties. The examination of footprint measurements is important in developing countries (e.g. Bangladesh, India, and Pakistan), because people walk barefoot for socioeconomic reasons. Footprints are present at the locations of crimes, such as theft and murder; footprints can be found on mud, dust, cement, oil, and painted surfaces, as well as in blood during murder cases. Therefore, analyses of feet and footprints during stature estimation can be useful in identification of criminals [1–6]. Previously, researchers have attempted to estimate human stature from feet and footprints in various populations. For instance, Kanchan et al. [3] conducted research on footprints and their components for stature estimation among Indian individuals. Krishan [7] performed a study involving 1 040 Gujjar men and 1 040 Gujjar women in northern India, aged 18–30 years, for stature determination. Hemy et al. [2] estimated stature using anthropometry of foot and footprint data from 200 adults (90 men, 110 women) in Western Australia. Other stature estimation studies using footprints were performed by Fawzy and Kamal [1] in Egyptian individuals, Abledu et al. [6] in Ghanaian individuals, Caplova et al. [8] in Slovak adults, and Khan and Nataraja Moorthy [9] in indigenous individuals from Malaysian Borneo.

The measurement of individuals varies among populations [10, 11]. Therefore, models to estimate stature are population-specific [10, 12]; a single model cannot represent all populations worldwide [9, 13, 14]. In Bangladesh, Asadujjaman et al. [15] examined foot measurements to estimate the stature of unknown individuals. However, to the best of our knowledge, there is no established standard formula to estimate stature from footprints in Bangladeshi individuals. Therefore, the present study was performed to investigate the relationship of stature with footprint measurements, and to develop a standard model for estimating the stature of Bangladeshi adults. This study used linear regression analysis for stature estimation, because this method exhibits considerable accuracy [9, 16].

## Materials and methods

### Materials

This study included 248 adults (118 men and 130 women) without any physical disorders. Previous studies [17] have shown that increasing age is associated with smaller foot dimensions. Moreover, the thickness and cross-sectional area of most foot muscles are significantly smaller in older adults specially over 50 years old [18], as is abductor hallucis muscle size [17]. Therefore, participants selected for this study were within the age range of 18–50 years. Data for this study were collected from different regions of Bangladesh between May 2018 and August 2018; data collection was performed between 10:00 am and 2:00 pm. This research was performed in a manner that protected the personal information of each participant.

### Method

**Figure 1.**
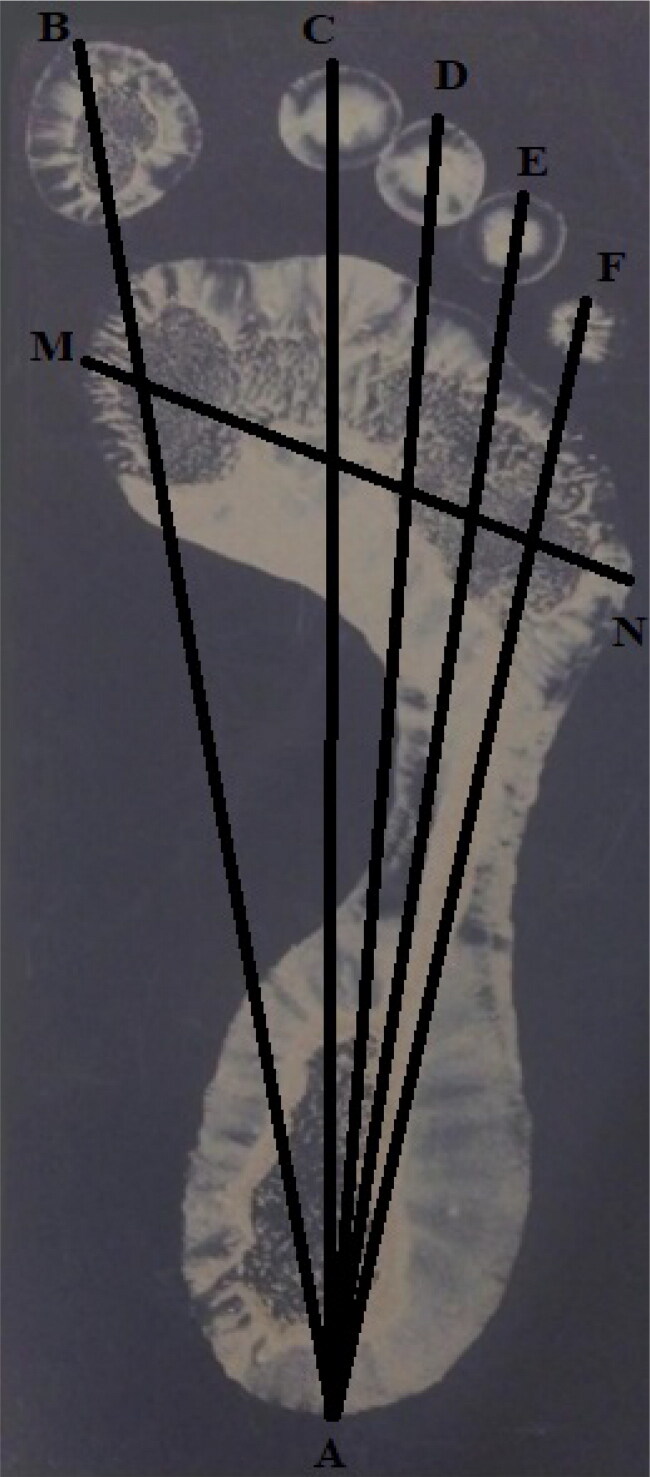
Footprint measurements: AB: T1 toe-print length; AC: T2 toe-print length; AD: T3 toe-print length; AE: T4 toe-print length; AF: T5 toe-print length; MN: footprint breadth at ball.

**Figure 2.**
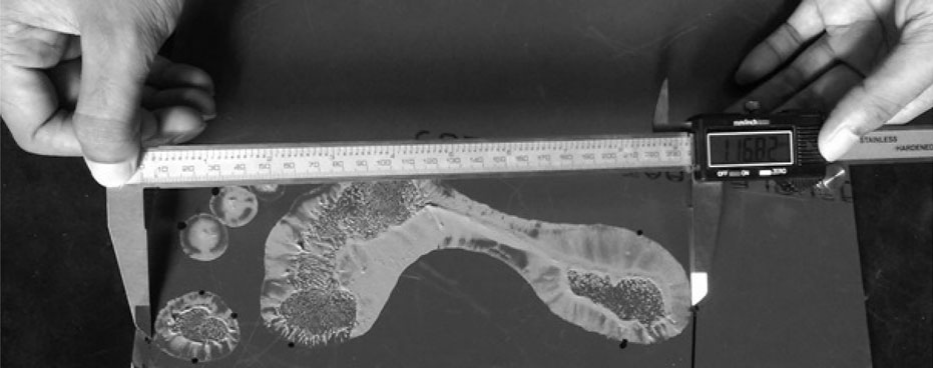
Footprint measurement technique.

In this study, a standard measuring tape and a digital slide calliper were used for a single stature measurement and six footprint measurements, including five toe-print lengths (named T1, T2, T3, T4, and T5) and footprint breadth at ball (FPBB). All measurements were performed in accordance with the methods of Hemy et al. [2] and Kanchan et al. [3]. Landmarks of the various footprint measurements are shown in Figure 1; Figure 2 presents the technique used to take footprint measurements.

Stature is the natural height of a person in an upright position [2, 3]. The toe-print lengths T1, T2, T3, T4, and T5 are the distance from the mid-rear heel point (A) to the most anterior point of each toe (B, C, D, E and F, respectively; Figure 1) [2, 3]. The FPBB was measured from the metatarsal lateral (N), the most lateral point of the metatarsophalangeal joint of T5, to the metatarsal medial (M), the most medial point of the metatarso-phalangeal joint of T1 [2].

Static footprints were obtained from the right and left feet of all participants. A novel method was used for collection of footprint measurements. Initially, participants were asked to walk over muddy soil in bare feet; they were then asked to stand on a clean glass plate, thus capturing the footprint on the glass plate. Subsequently, a marker pen was used to mark some points on the feet for use in footprint measurements. Finally, footprint measurements were taken using digital callipers. All the measurements were taken by one observer to avoid the interobserver error. Measurements were taken from both right and left footprints. Each measurement was taken two times; when measurements difference remained within 0.4 mm, the average value was recorded to minimise error. If the two preliminary measurements did not agree the 0.4-mm threshold criterion, two additional measurements were taken, and the average value of the second set of measurement was recorded.

### Statistical analysis

Statistical analyses were performed using Microsoft Excel 2013 (Microsoft Corp., Redmond, WA, USA) and SPSS Statistics, version 23.0 (IBM Corp., Armonk, NY, USA). The normality of the sample data was assessed using the Shapiro-Wilk test, prior to selection of parametric or non-parametric comparisons. Sex-based comparisons were conducted using independent *t*-tests and the non-parametric alternative, the Mann–Whitney *U* test. Differences between two groups were assessed using paired sample *t*-tests and the non-parametric alternative, the Wilcoxon signed-rank test. Linear regression analysis was used to estimate stature from footprint anthropometric measurements. *P* < 0.05 was considered to be statistically significant.

## Results

### Normality tests

**Figure 3.**
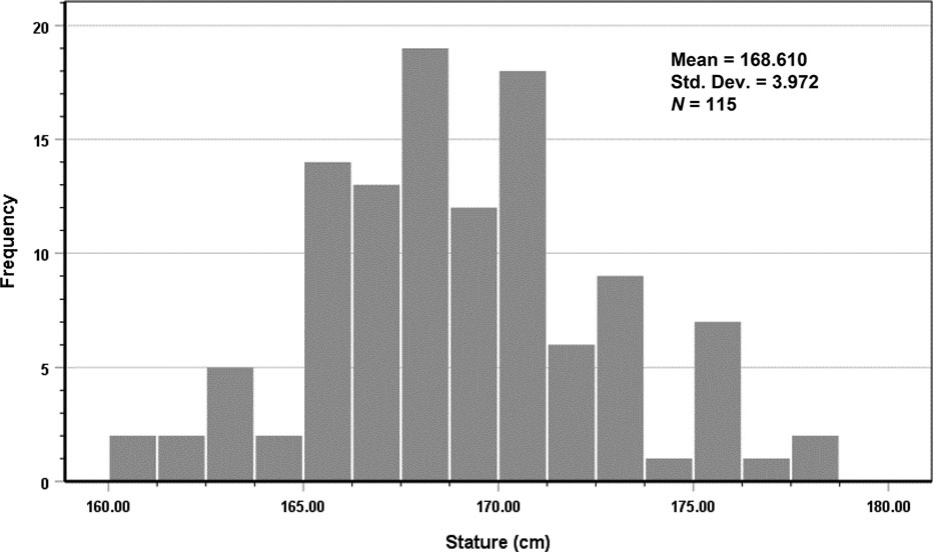
Histogram of stature in men.

**Figure 4.**
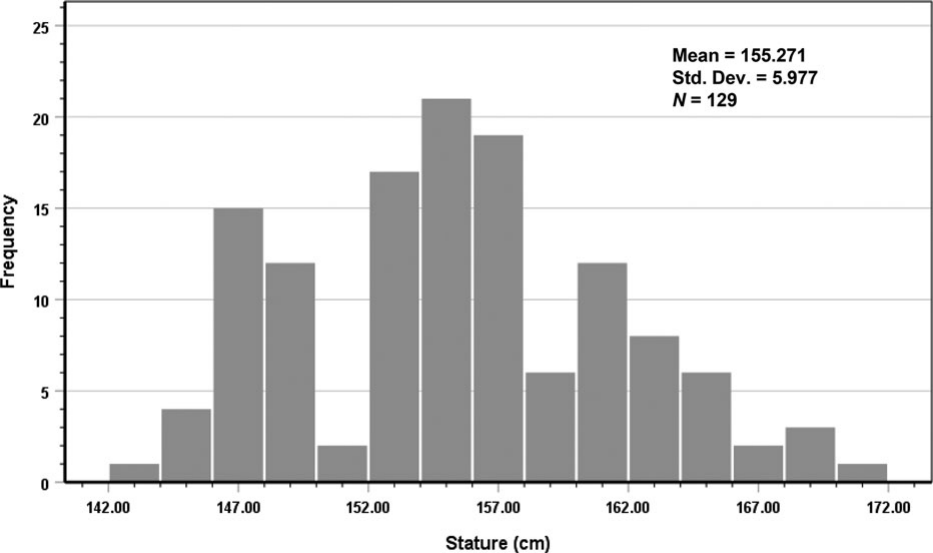
Histogram of stature in women.

Normality tests were performed on stature and other footprint measurements; all measurements were evaluated separately for men and women. The Shapiro–Wilk test showed that the data for both men and women did not exhibit normal distributions (all *P* < 0.05). After removal of three outlier men and one outlier woman, the Shapiro–Wilk test indicated that the sample data demonstrated a normal distribution (all *P* > 0.05). Therefore, the remaining analyses included 115 men and 129 women. Histograms that demonstrate stature distributions of men and women are shown in Figures 3 and 4, respectively.

Normality tests of stature and footprint measurement data (shown in Table 1) revealed that the right T1 lengths were not normally distributed in either men or women; right T2 length, right T3 length, left T2 length, left T3 length, and left FPBB were not normally distributed in women; right T5 length, right FPBB, left T1 length, and left T5 length were not normally distributed in men (all *P* < 0.05). Hence, the non-parametric Mann–Whitney *U* test was used for those footprint measurements to examine sexual dimorphism.

**Table 1.** Normality tests (Shapiro–Wilk) for stature and footprint measurements according to sex (men: *n* = 115; women: *n* = 129).

Measurement	Sex	Statistic	*P*-value
Stature	Women	0.980	0.051
	Men	0.983	0.148
Right T1	Women	0.971	0.007^a^
	Men	0.963	0.003^a^
Right T2	Women	0.965	0.002^a^
	Men	0.983	0.170
Right T3	Women	0.974	0.016^a^
	Men	0.981	0.101
Right T4	Women	0.993	0.819
	Men	0.989	0.521
Right T5	Women	0.990	0.495
	Men	0.958	0.001^a^
Right FPBB	Women	0.994	0.843
	Men	0.959	0.001^a^
Left T1	Women	0.984	0.131
	Men	0.972	0.018^a^
Left T2	Women	0.965	0.002^a^
	Men	0.991	0.624
Left T3	Women	0.978	0.033^a^
	Men	0.988	0.423
Left T4	Women	0.986	0.208
	Men	0.987	0.340
Left T5	Women	0.984	0.132
	Men	0.959	0.001^a^
Left FPBB	Women	0.978	0.037^a^
	Men	0.981	0.103

T1, T2, T3, T4, T5: the distance from the mid-rear heel point to the most anterior point of the thumb toe, index toe, middle toe, fourth toe, and little toe, respectively; FPBB: footprint breadth at ball.

^a^ Denotes absence of normality for the indicated measurement (*P* < 0.05).

Normality tests for footprint measurements by side (Table 2) revealed that T1 length (both sides), T5 length (both sides), and FPBB (right side) did not exhibit normal distributions (all *P* < 0.05) in men. In women, T1 length (right side), T2 length (both sides), T3 length (right side), and FPBB (left side) did not exhibit normal distributions (all *P* < 0.05). Therefore, the non-parametric Wilcoxon signed rank test was used for those footprint measurements to examine bilateral asymmetry.

**Table 2.** Normality tests (Shapiro–Wilk) for footprint measurements according to side.

Measurement	Side	Men (*n* = 115)		Women (*n* = 129)	
		Statistic	*P*	Statistic	*P*
T1	Left	0.972	0.018^a^	0.984	0.131
	Right	0.963	0.003^a^	0.971	0.007^a^
T2	Left	0.991	0.625	0.965	0.002^a^
	Right	0.983	0.170	0.965	0.002^a^
T3	Left	0.988	0.423	0.978	0.033
	Right	0.981	0.100	0.974	0.016^a^
T4	Left	0.987	0.341	0.986	0.209
	Right	0.989	0.521	0.993	0.819
T5	Left	0.959	0.001^a^	0.984	0.132
	Right	0.958	0.001^a^	0.990	0.495
FPBB	Left	0.981	0.104	0.978	0.037^a^
	Right	0.959	0.001^a^	0.994	0.843

T1, T2, T3, T4, T5: the distance from the mid-rear heel point to the most anterior point of the thumb toe, index toe, middle toe, fourth toe, and little toe, respectively; FPBB: footprint breadth at ball.

^a^ Denotes absence of normality for the indicated measurement (*P* < 0.05).

### Significance test

Descriptive statistics, including maximum value, minimum value, and mean and standard deviation values for footprint dimensions, are presented in Table 3. All footprint dimensions were larger in men than in women. When stature and footprint measurement data were compared according to sex (Tables 4 and 5), significant (*P* < 0.001) sexual dimorphism was observed in all measurements.

**Table 3.** Descriptive statistics for stature and footprint measurements, according to sex.

Parameter	Men (*n* = 115)				Women (*n* = 129)			
	Min	Max	Mean	SD	Min	Max	Mean	SD
Stature	157.48	177.80	168.61	3.97	142.24	171.00	155.27	5.98
Right T1	21.53	25.34	23.83	0.75	19.75	23.98	22.12	0.92
Right T2	21.65	26.66	23.81	0.87	19.45	23.89	21.88	0.94
Right T3	21.03	24.62	22.88	0.79	18.22	23.00	21.01	0.97
Right T4	20.11	23.57	21.83	0.77	17.62	22.53	19.93	0.97
Right T5	18.69	22.83	20.36	0.64	16.07	20.49	18.39	0.90
Right FPBB	7.65	10.41	9.28	0.50	6.87	9.81	8.43	0.55
Left T1	21.46	25.56	23.88	0.78	19.93	24.40	22.20	0.93
Left T2	21.69	26.24	23.86	0.86	19.54	24.00	21.93	0.96
Left T3	20.93	24.76	22.94	0.81	18.46	23.02	21.04	0.97
Left T4	20.06	23.61	21.85	0.79	17.61	22.50	19.89	0.99
Left T5	18.77	22.64	20.39	0.68	16.16	22.21	18.45	0.99
Left FPBB	7.67	10.47	9.25	0.52	7.09	9.84	8.45	0.53

T1, T2, T3, T4, T5: the distance (cm) from the mid-rear heel point to the most anterior point of the thumb toe, index toe, middle toe, fourth toe, and little toe, respectively; FPBB: footprint breadth (cm) at ball.

**Table 4.** Independent *t*-test of means for stature and footprint measurements, according to sex.

Measurement	*t*	*P* (2-tailed)
Stature	−20.269	0.000^a^
Right T4	−16.739	0.000^a^
Left T4	−16.929	0.000^a^

^a^ Significant (*P* < 0.001).

**Table 5.** Mann-Whitney *U* of means for stature and footprint measurements, according to sex.

Mann-Whitney *U* test		
Measurement	Mann-Whitney *U*	*P* (2-tailed)
Right T1	1045.0	0.000^a^
Right T2	888.0	0.000^a^
Right T3	978.0	0.000^a^
Right T5	403.5	0.000^a^
Right FPBB	1896.5	0.000^a^
Left T1	1193.5	0.000^a^
Left T2	854.0	0.000^a^
Left T3	938.0	0.000^a^
Left T5	644.5	0.000^a^
Left FPBB	2010.5	0.000^a^

T1, T2, T3, T4, T5: the distance from the mid-rear heel point to the most anterior point of the thumb toe, index toe, middle toe, fourth toe, and little toe, respectively; FPBB: footprint breadth at ball.

^a^ Significant (*P* < 0.001).

Bilateral asymmetry analyses of footprint measurements (Table 6) revealed that T1, T2, and T3 lengths were significantly different in men, whereas the T4 and T5 lengths and the FPBB were not. Furthermore, the T1 and T2 lengths were significantly different in women, whereas the T3, T4, and T5 lengths and the FPBB were not.

**Table 6.** Comparison of means for footprint measurements, according to side.

Sex	Test	Pair	*t*/*z*-value^a^	*P*-value (2-tailed)
Men	Paired sample *t*-test	Right-Left T2	−2.571	0.011^b^
		Right-Left T3	−2.820	0.006^b^
		Right-Left T4	−1.002	0.318
	Wilcoxon signed rank test	Right-Left T1	−3.094	0.002^b^
		Right-Left T5	−1.853	0.064
		Right-Left FPBB	−0.717	0.473
Women	Paired sample *t*-test	Right-Left T4	1.285	0.201
		Right-Left T5	−1.454	0.148
	Wilcoxon signed rank test	Right-Left T1	−3.207	0.001^b^
		Right-Left T2	−2.018	0.044^b^
		Right-Left T3	−1.744	0.081
		Right-Left FPBB	−1.151	0.250

T1, T2, T3, T4, T5: the distance from the mid-rear heel point to the most anterior point of thumb toe, index toe, middle toe, fourth toe, and little toe, respectively; FPBB: footprint breadth at ball.

^a^ *t* for paired sample *t*-test; *z* for Wilcoxon signed rank test.

^b^ Significant (*P* < 0.05).

### Linear regression analysis

Linear regression models to estimate stature based on right and left footprint dimensions in both sexes are presented in Table 7. Investigators and police did not know whether a footprint was made by a man or a woman; therefore, regression equations were developed by combining data for both men and women. Values of the coefficient of correlation (*R*), coefficient of determination (*R^2^*), standard error of estimate (SEE), and 95% prediction interval are also shown in Table 7. Values of *R* varied from +0.380 to +0.587 in men and from +0.193 to +0.506 in women. Regression equations developed by combining data for both men and women revealed a higher value of *R*, indicating that the largest value of *R* was found between stature and right T2 length (+0.792) and the lowest value was found between stature and left FPBB (+0.606).

**Table 7.** Linear regression equations for stature estimation (cm) from footprint measurements.

Sex	Side	Equation	*R*	*R^2^*	SEE	95% prediction interval	*P*
Men (*n* = 115)	Right	S = 94.412 + 3.114 T1	0.587	0.345	3.230	6.331	0.000^a^
	Left	S = 101.996 + 2.789 T1	0.550	0.302	3.333	6.533	0.000^a^
	Right	S = 109.279 + 2.492 T2	0.547	0.299	3.340	6.546	0.000^a^
	Left	S = 107.850 + 2.547 T2	0.549	0.302	3.333	6.533	0.000^a^
	Right	S = 108.304 + 2.636 T3	0.523	0.274	3.400	6.664	0.000^a^
	Left	S = 109.498 + 2.577 T3	0.525	0.276	3.396	6.656	0.000^a^
	Right	S = 112.577 + 2.567 T4	0.499	0.249	3.457	6.776	0.000^a^
	Left	S = 115.481 + 2.434 T4	0.485	0.235	3.489	6.838	0.000^a^
	Right	S = 102.931 + 3.225 T5	0.522	0.273	3.402	6.668	0.000^a^
	Left	S = 107.067 + 3.019 T5	0.519	0.270	3.410	6.684	0.000^a^
	Right	S = 140.762 + 3.001 FPBB	0.380	0.145	3.690	7.232	0.000^a^
	Left	S = 141.212 + 2.960 FPBB	0.388	0.151	3.677	7.207	0.000^a^
Women (*n* = 129)	Right	S = 87.810 + 3.050 T1	0.472	0.223	5.291	10.370	0.000^a^
	Left	S = 85.621 + 3.137 T1	0.489	0.239	5.236	10.263	0.000^a^
	Right	S = 85.171 + 3.204 T2	0.506	0.256	5.177	10.147	0.000^a^
	Left	S = 90.573 + 2.951 T2	0.475	0.225	5.282	10.353	0.000^a^
	Right	S = 102.341 + 2.519 T3	0.411	0.169	5.472	10.725	0.000^a^
	Left	S = 104.093 + 2.432 T3	0.397	0.157	5.506	10.792	0.000^a^
	Right	S = 110.502 + 2.246 T4	0.366	0.134	5.585	10.947	0.000^a^
	Left	S = 110.318 + 2.260 T4	0.374	0.140	5.565	10.907	0.000^a^
	Right	S = 108.695 + 2.532 T5	0.382	0.146	5.545	10.868	0.000^a^
	Left	S = 118.351 + 2.001 T5	0.333	0.111	5.659	11.092	0.000^a^
	Right	S = 132.905 + 2.748 FPBB	0.252	0.064	5.806	11.380	0.000^a^
	Left	S = 137.090 + 2.151 FPBB	0.193	0.037	5.889	11.542	0.000^a^
Combined (*n* = 244)	Right	S = 36.242 + 5.467 T1	0.781	0.610	5.260	10.310	0.000^a^
	Left	S = 37.310 + 5.404 T1	0.775	0.600	5.331	10.449	0.000^a^
	Right	S = 47.027 + 5.026 T2	0.792	0.627	5.144	10.082	0.000^a^
	Left	S = 48.143 + 4.996 T2	0.784	0.614	5.234	10.259	0.000^a^
	Right	S = 52.982 + 4.960 T3	0.762	0.580	5.459	10.700	0.000^a^
	Left	S = 54.382 + 4.886 T3	0.758	0.575	5.494	10.768	0.000^a^
	Right	S = 60.248 + 4.865 T4	0.750	0.562	5.579	10.935	0.000^a^
	Left	S = 62.510 + 4.759 T4	0.752	0.566	5.553	10.884	0.000^a^
	Right	S = 61.143 + 5.197 T5	0.780	0.609	5.269	10.327	0.000^a^
	Left	S = 67.275 + 4.869 T5	0.749	0.562	5.580	10.937	0.000^a^
	Right	S = 91.618 + 7.919 FPBB	0.637	0.405	6.450	12.642	0.000^a^
	Left	S = 93.597 + 7.696 FPBB	0.606	0.368	6.702	13.136	0.000^a^

T1, T2, T3, T4, T5: the distance from the mid-rear heel point to the most anterior point of thumb toe, index toe, middle toe, fourth toe, and little toe, respectively; FPBB: footprint breadth at ball; SEE: standard error of estimate.

^a^ Significant (*P* < 0.001).

Values of *R^2^* varied from 0.145 to 0.345 in men, 0.037 to 0.256 in women, and 0.368 to 0.627 in the combined dataset. The SEE ranged from ±3.230 to ±3.690 cm in men, ±5.177 to ±5.889 cm in women, and ±5.144 to ±6.702 cm in the combined dataset. The 95% prediction interval ranged from ±6.331 to ±7.232 cm in men, ±10.147 to ±11.542 cm in women, and ±10.082 to ±13.136 cm in the combined dataset. All footprint measurements were significantly associated (*P* < 0.001) with stature.

**Figure 5.**
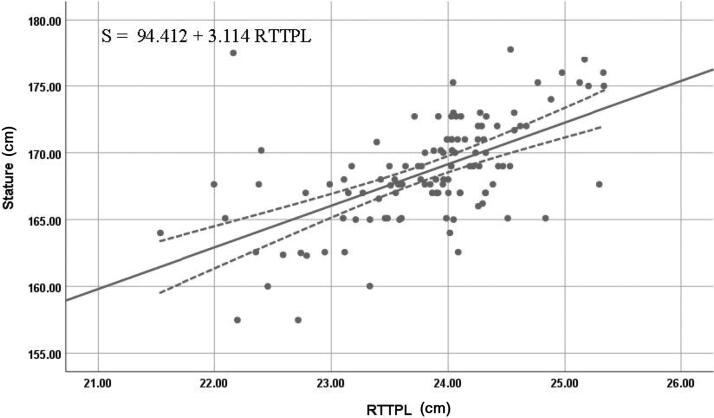
Best fit curve to estimate stature from right T1 length in men. RTTPL: right thumb toe-print length. The straight line indicates the best fit line and the dotted line indicates the 95% prediction interval.

**Figure 6.**
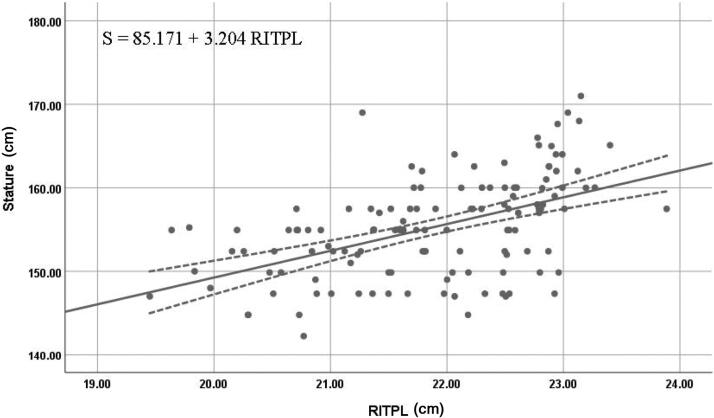
Best fit curve to estimate stature from right T2 length in women. RITPL: right index toe-print length. The straight line indicates the best fit line and the dotted line indicates the 95% prediction interval.

**Figure 7.**
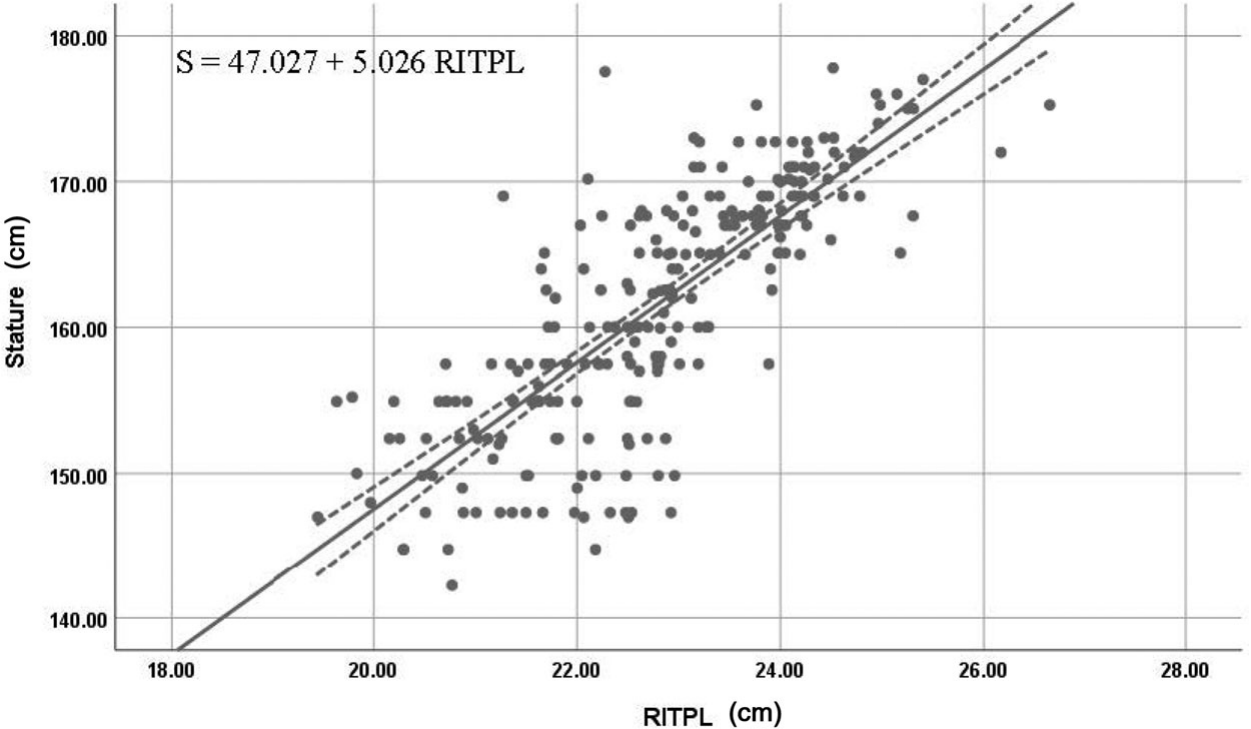
Best fit curve to estimate stature from right T2 length using combined data (from both sexes). RITPL: right index toe-print length. The straight line indicates the best fit line and the dotted line indicates the 95% prediction interval.

The right T1 length in men and right T2 lengths in both women and the combined dataset were the most reliable individual estimators of stature; they exhibited high values of *R* and *R^2^*, along with low values of SEE and 95% prediction interval. Figures 5–7 show the best fit curves of the most reliable estimators of stature for men, women, and combined data, respectively.

## Discussion

In this study, significant bilateral asymmetry was found for the T1, T2, and T3 lengths in men, as well as for the T1 and T2 lengths in women (Table 6). Previously, Kanchan et al. [3] found significant bilateral asymmetry for the T1 and T2 lengths in Indian men, as well as for the T1, T2, T3, T4, and T5 lengths in Indian women. Khan and Nataraja Moorthy [9] did not find significant (*P* > 0.05) bilateral asymmetry for footprint measurements among indigenous Melanaus men in Malaysian Borneo; however, they found that the right-left side differences for the T1 and T2 lengths were statistically significant (*P* < 0.01) in women. Moreover, Krishan et al. [7] found significant (*P* < 0.01) bilateral asymmetry for the T2 and T4 lengths among Gujjar men in North India.

In our study, the combined data exhibited greater accuracy in stature estimation from footprint measurements (Table 7). The right T2 length was the most reliable estimator when combined data were used (*R*: 0.792; *R^2^*: 0.627). Tables 8 and 9 show comparative analyses of the values of *R* and *R^2^* between stature and different footprint measurements among various populations. In the present study, all footprint measurements were positively correlated with stature. In previous studies, the values of *R* between stature and a variety of footprint dimensions were also positive (Table 8). In the present study, the right T1 length in men and right T2 length in women were the most reliable individual estimators of stature, with higher values of *R* (0.587 in men and 0.506 in women) and *R^2^* (0.345 in men and 0.256 in women). In the study of Egyptian men by Fawzy et al. [1], higher values of *R* (0.570) and *R^2^* (0.332) were found between stature and right T5 length. In a Western Australian population [2], the left T2 length in men and right T1 length in women were the most reliable estimators (*R*: 0.728 in men and 0.716 in women). In an Indian population [3], the left T1 length was the most reliable individual estimator in men (*R*: 0.628, *R^2^*: 0.395); in Indian women, higher values of *R* (0.527) and *R^2^* (0.278) were found between right T1 length and stature. In the Melanaus indigenous population of Malaysian Borneo [9], the left T2 length in men and right T1 length in women were the most reliable estimators of stature (*R*: 0.789 in men and 0.830 in women; *R^2^*: 0.623 in men and 0.690 in women).

**Table 8.** Comparison of *R* values between stature and different footprint measurements among various populations.

Measurements	Present study				Fawzy et al. [1]		Hemy et al. [2]				Kanchan et al. [3]				Khan and Nataraja Moorthy [9]			
	Men		Women		Men		Men		Women		Men		Women		Men		Women	
	Right	Left	Right	Left	Right	Left	Right	Left	Right	Left	Right	Left	Right	Left	Right	Left	Right	Left
T1	0.587	0.550	0.472	0.489	0.540	0.540	0.678	0.706	0.716	0.694	0.628	0.628	0.527	0.444	0.726	0.766	0.830	0.818
T2	0.547	0.549	0.506	0.475	0.400	0.430	0.668	0.728	0.646	0.659	0.600	0.584	0.504	0.449	0.734	0.789	0.811	0.807
T3	0.523	0.525	0.411	0.397	0.410	0.400	0.666	0.703	0.643	0.662	0.556	0.561	0.421	0.407	0.739	0.771	0.793	0.795
T4	0.499	0.485	0.366	0.374	0.440	0.410	0.684	0.723	0.650	0.684	0.577	0.573	0.466	0.428	0.729	0.756	0.801	0.805
T5	0.522	0.519	0.382	0.333	0.570	0.520	0.652	0.678	0.701	0.684	0.504	0.464	0.522	0.451	0.707	0.760	0.777	0.774
FPBB	0.380	0.388	0.252	0.193	0.040	0.210	0.427	0.507	0.296	0.259	–	–	–	–	–	–	–	–

T1, T2, T3, T4, T5: the distance from the mid-rear heel point to the most anterior point of thumb toe, index toe, middle toe, fourth toe, and little toe, respectively; FPBB: footprint breadth at ball.

**Table 9.** Comparison of *R^2^* values between stature and different footprint measurements among various populations.

Measurements	Present study				Fawzy et al. [1]		Kanchan et al. [3]				Khan and Nataraja Moorthy [9]			
	Men		Women		Men		Men		Women		Men		Women	
	Right	Left	Right	Left	Right	Left	Right	Left	Right	Left	Right	Left	Right	Left
T1	0.345	0.302	0.223	0.239	0.293	0.290	0.394	0.395	0.278	0.197	0.528	0.587	0.690	0.669
T2	0.299	0.302	0.256	0.225	0.162	0.186	0.360	0.341	0.254	0.201	0.539	0.623	0.658	0.652
T3	0.274	0.276	0.169	0.157	0.173	0.159	0.309	0.314	0.178	0.165	0.546	0.594	0.629	0.632
T4	0.249	0.235	0.134	0.140	0.197	0.168	0.333	0.329	0.217	0.183	0.531	0.571	0.641	0.648
T5	0.273	0.270	0.146	0.111	0.332	0.271	0.254	0.216	0.272	0.203	0.500	0.587	0.604	0.599
FPBB	0.145	0.151	0.064	0.037	0.094	0.064	–	–	–	–	–	–	–	–

T1, T2, T3, T4, T5: the distance from the mid-rear heel point to the most anterior point of thumb toe, index toe, middle toe, fourth toe, and little toe, respectively; FPBB: footprint breadth at ball.

Table 10 shows a comparison of studies regarding stature estimation from footprint measurements, using simple linear regression analysis. In the present study, lower values of SEE were able to estimate stature, using linear regression models. The values of SEE varied from ±3.230 to ±3.690 cm in men, ±5.177 to ±5.889 cm in women, and ±5.144 to ±6.702 cm in the combined data. In Egyptian men [1], SEE varied from ±3.52 to ±4.69 cm. In a Western Australian population [2], the values of SEE varied from ±4.885 to ±6.439 cm in men and ±5.006 to ±6.926 cm in women. In another Indian population [3], SEE varied between ±4.1088 and ±4.4470 cm in men and between ±5.2866 and ±5.6838 cm in women. In the Melanaus indigenous population of Malaysian Borneo [9], SEE varied from ±3.506 to ±4.037 cm in men and ±3.316 to ±3.785 cm in women.

**Table 10.** Comparison of results among studies concerning stature estimation from footprint measurements *via* linear regression.

Study	Sample size	Ages (years)	Population	Parameters	SEE
Present study	115 men and 129 women	18 to 50	Bangladesh	T1, T2, T3, T4, T5, FPBB	±3.230 to ±3.690 cm in men and ±5.177 to ±5.889 cm in women, ±5.144 to ±6.702 cm in combined data
Fawzy et al. [1]	50 men	18 to 25	Egypt	T1, T2, T3, T4, T5, FPBB, breadth at heel, big toe pad length, big toe pad breadth	±3.52 to ±4.69 cm in men
Hemy et al. [2]	90 men and 110 women	19 to 68 in men and 18 to 63 in women	Western Australia	T1, T2, T3, T4, T5, FPBB, breadth at heel	±4.885 to ±6.439 cm in men and ±5.006 to ±6.926 cm in women
Kanchan et al. [3]	50 men and 50 women	20 to 25	India	T1, T2, T3, T4, T5	±4.1088 to ±4.4470 cm in men and ±5.2866 to ±5.6838 cm in women
Khan and Nataraja Moorthy [9]	105 men and 105 women	18 to 59	Melanaus indigenous population of Malaysian Borneo	T1, T2, T3, T4, T5	±3.506 to ±4.037 cm in men and ±3.316 to ±3.785 cm in women

T1, T2, T3, T4, T5: the distance from the mid-rear heel point to the most anterior point of thumb toe, index toe, middle toe, fourth toe, and little toe, respectively; FPBB: footprint breadth at ball; SEE: standard error of estimate.

## Conclusion

This study developed regression formulae to estimate stature from footprint measurements in Bangladeshi adults. The present investigation revealed that human stature can be estimated with reasonably accuracy from footprint measurements in the Bangladeshi population. The findings of this study will be useful in forensic research and crime investigation by law enforcement agencies. The age range of this study was large; therefore, future studies should be performed in specific age groups.
